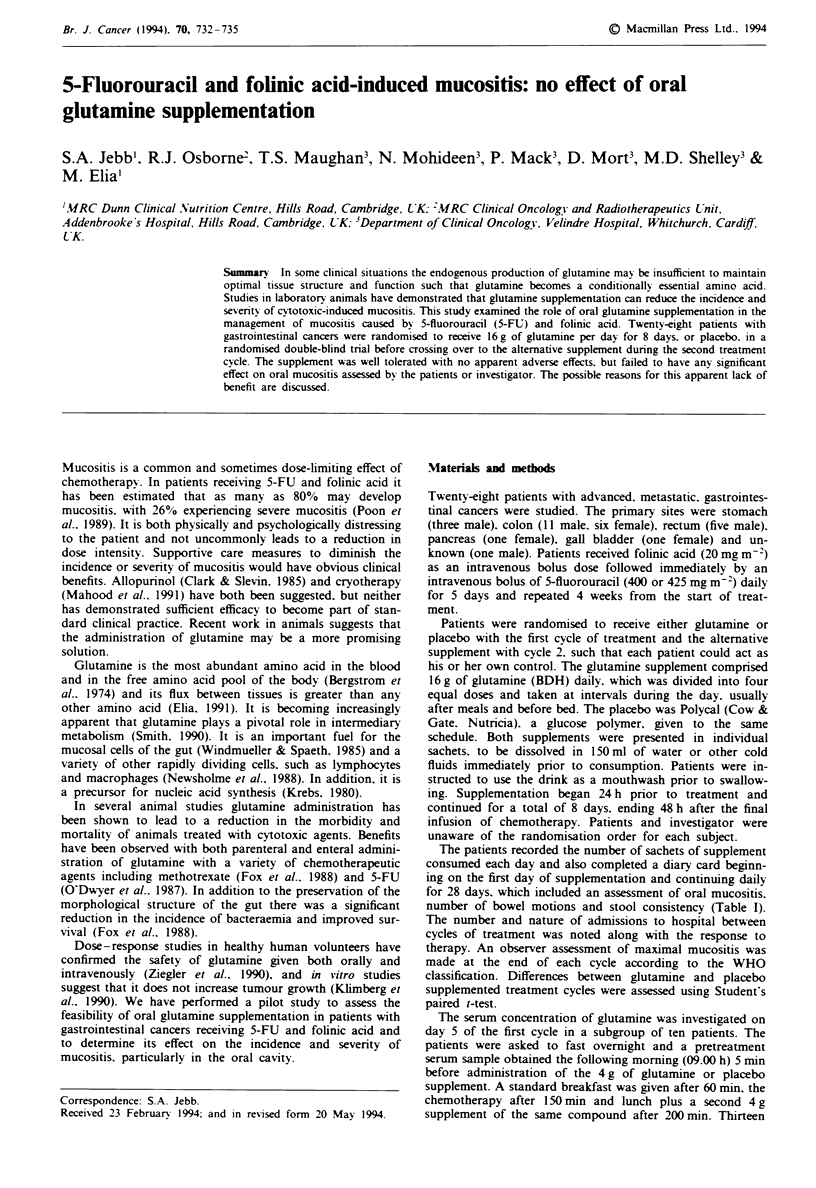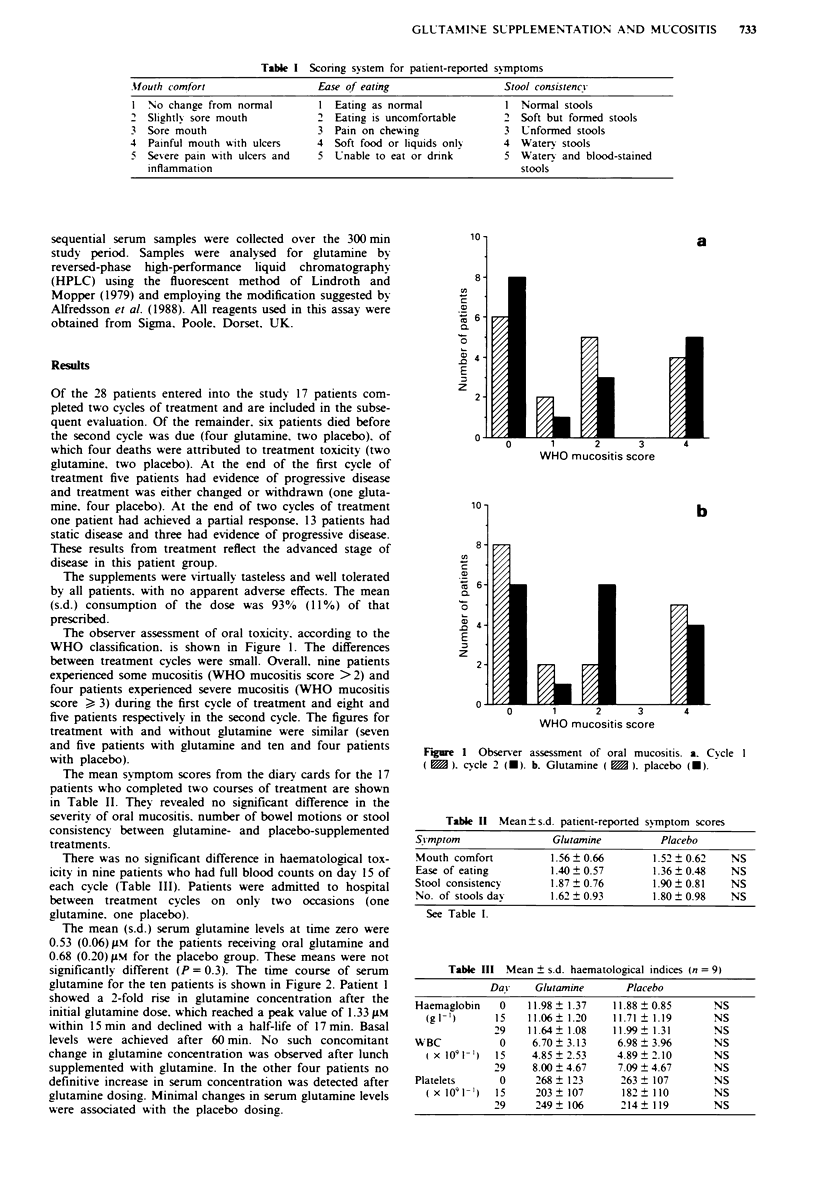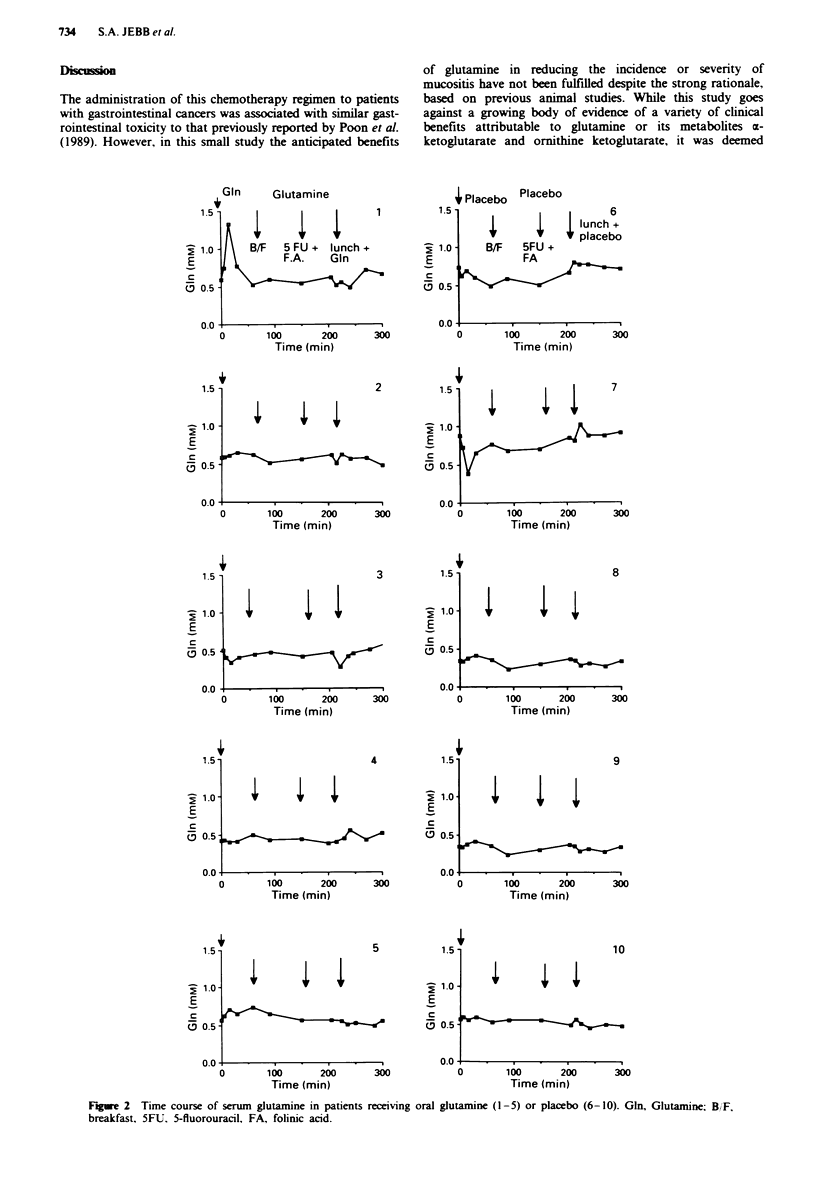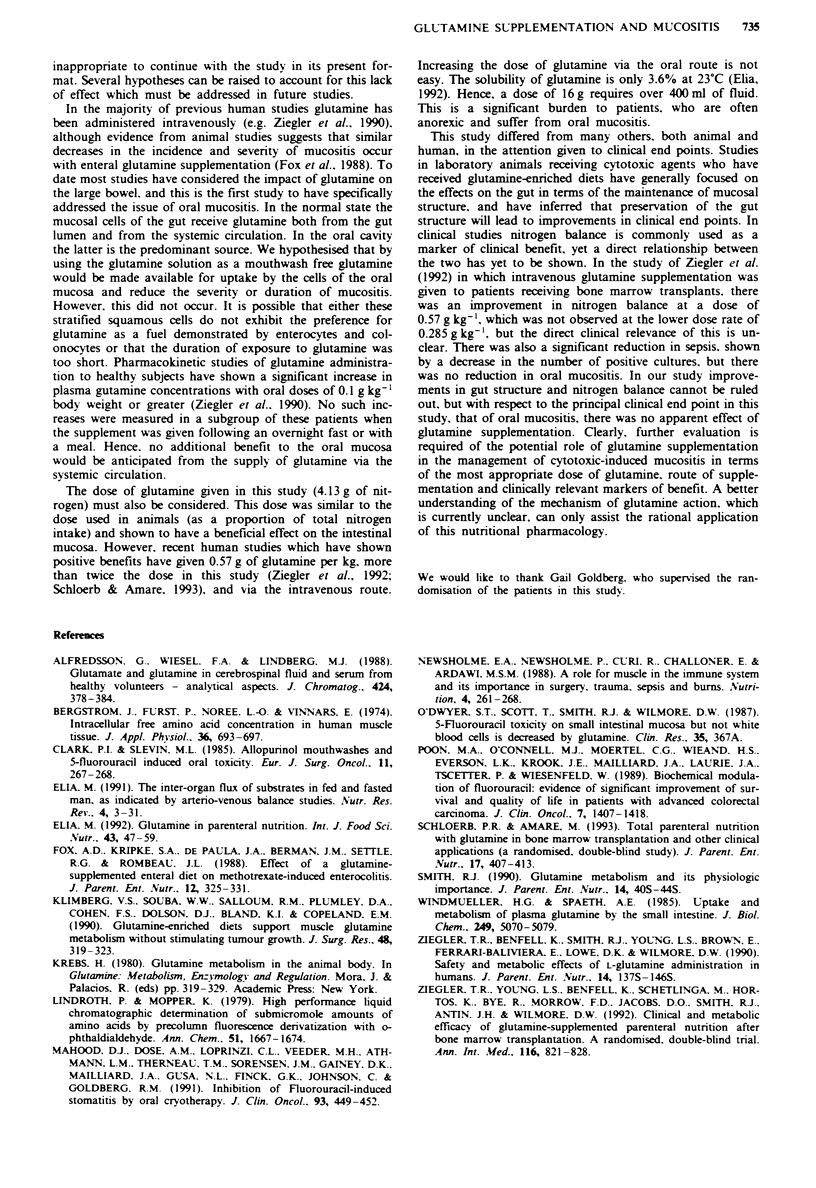# 5-fluorouracil and folinic acid-induced mucositis: no effect of oral glutamine supplementation.

**DOI:** 10.1038/bjc.1994.385

**Published:** 1994-10

**Authors:** S. A. Jebb, R. J. Osborne, T. S. Maughan, N. Mohideen, P. Mack, D. Mort, M. D. Shelley, M. Elia

**Affiliations:** MRC Dunn Clinical Nutrition Centre, Cambridge, UK.

## Abstract

In some clinical situations the endogenous production of glutamine may be insufficient to maintain optimal tissue structure and function such that glutamine becomes a conditionally essential amino acid. Studies in laboratory animals have demonstrated that glutamine supplementation can reduce the incidence and severity of cytotoxic-induced mucositis. This study examined the role of oral glutamine supplementation in the management of mucositis caused by 5-fluorouracil (5-FU) and folinic acid. Twenty-eight patients with gastrointestinal cancers were randomised to receive 16 g of glutamine per day for 8 days, or placebo, in a randomised double-blind trial before crossing over to the alternative supplement during the second treatment cycle. The supplement was well tolerated with no apparent adverse effects, but failed to have any significant effect on oral mucositis assessed by the patients or investigator. The possible reasons for this apparent lack of benefit are discussed.


Br. J. Cancer (1994), 70, 732-735 © Macmillan Press Ltd., 1994

### 5-Fluorouracil and folinic acid-induced mucositis: no effect of oral glutamine supplementation

S.A. Jebb[1], R.J. Osborne[2], T.S. Maughan[3], N. Mohideen[3], P. Mack[3], D. Mort[3], M.D. Shelley[3] & M. Elia[1]

[1]*MRC Dunn Clinical Nutrition Centre, Hills Road, Cambridge, UK;* [2]*MRC Clinical Oncology and Radiotherapeutics Unit, Addenbrooke's Hospital, Hills Road, Cambridge, UK;* [3]*Department of Clinical Oncology, Velindre Hospital, Whitchurch, Cardiff, UK.*

**Summary** In some clinical situations the endogenous production of glutamine may be insufficient to maintain optimal tissue structure and function such that glutamine becomes a conditionally essential amino acid. Studies in laboratory animals have demonstrated that glutamine supplementation can reduce the incidence and severity of cytotoxic-induced mucositis. This study examined the role of oral glutamine supplementation in the management of mucositis caused by 5-fluorouracil (5-FU) and folinic acid. Twenty-eight patients with gastrointestinal cancers were randomised to receive 16 g of glutamine per day for 8 days, or placebo, in a randomised double-blind trial before crossing over to the alternative supplement during the second treatment cycle. The supplement was well tolerated with no apparent adverse effects, but failed to have any significant effect on oral mucositis assessed by the patients or investigator. The possible reasons for this apparent lack of benefit are discussed.

Mucositis is a common and sometimes dose-limiting effect of chemotherapy. In patients receiving 5-FU and folinic acid it has been estimated that as many as 80% may develop mucositis, with 26% experiencing severe mucositis (Poon *et al.*, 1989). It is both physically and psychologically distressing to the patient and not uncommonly leads to a reduction in dose intensity. Supportive care measures to diminish the incidence or severity of mucositis would have obvious clinical benefits. Allopurinol (Clark & Slevin, 1985) and cryotherapy (Mahood *et al.*, 1991) have both been suggested, but neither has demonstrated sufficient efficacy to become part of standard clinical practice. Recent work in animals suggests that the administration of glutamine may be a more promising solution.

Glutamine is the most abundant amino acid in the blood and in the free amino acid pool of the body (Bergstrom *et al.*, 1974) and its flux between tissues is greater than any other amino acid (Elia, 1991). It is becoming increasingly apparent that glutamine plays a pivotal role in intermediary metabolism (Smith, 1990). It is an important fuel for the mucosal cells of the gut (Windmueller & Spaeth, 1985) and a variety of other rapidly dividing cells, such as lymphocytes and macrophages (Newsholme *et al.*, 1988). In addition, it is a precursor for nucleic acid synthesis (Krebs, 1980).

In several animal studies glutamine administration has been shown to lead to a reduction in the morbidity and mortality of animals treated with cytotoxic agents. Benefits have been observed with both parenteral and enteral administration of glutamine with a variety of chemotherapeutic agents including methotrexate (Fox *et al.*, 1988) and 5-FU (O'Dwyer *et al.*, 1987). In addition to the preservation of the morphological structure of the gut there was a significant reduction in the incidence of bacteraemia and improved survival (Fox *et al.*, 1988).

Dose-response studies in healthy human volunteers have confirmed the safety of glutamine given both orally and intravenously (Ziegler *et al.*, 1990), and *in vitro* studies suggest that it does not increase tumour growth (Klimberg *et al.*, 1990). We have performed a pilot study to assess the feasibility of oral glutamine supplementation in patients with gastrointestinal cancers receiving 5-FU and folinic acid and to determine its effect on the incidence and severity of mucositis, particularly in the oral cavity.

Correspondence: S.A. Jebb.
Received 23 February 1994; and in revised form 20 May 1994.

### Materials and methods

Twenty-eight patients with advanced, metastatic, gastrointestinal cancers were studied. The primary sites were stomach (three male), colon (11 male, six female), rectum (five male), pancreas (one female), gall bladder (one female) and unknown (one male). Patients received folinic acid (20 mg $m^{-2}$) as an intravenous bolus dose followed immediately by an intravenous bolus of 5-fluorouracil (400 or 425 mg $m^{-2}$) daily for 5 days and repeated 4 weeks from the start of treatment.

Patients were randomised to receive either glutamine or placebo with the first cycle of treatment and the alternative supplement with cycle 2, such that each patient could act as his or her own control. The glutamine supplement comprised 16 g of glutamine (BDH) daily, which was divided into four equal doses and taken at intervals during the day, usually after meals and before bed. The placebo was Polycal (Cow & Gate, Nutricia), a glucose polymer, given to the same schedule. Both supplements were presented in individual sachets, to be dissolved in 150 ml of water or other cold fluids immediately prior to consumption. Patients were instructed to use the drink as a mouthwash prior to swallowing. Supplementation began 24 h prior to treatment and continued for a total of 8 days, ending 48 h after the final infusion of chemotherapy. Patients and investigator were unaware of the randomisation order for each subject.

The patients recorded the number of sachets of supplement consumed each day and also completed a diary card beginning on the first day of supplementation and continuing daily for 28 days, which included an assessment of oral mucositis, number of bowel motions and stool consistency (Table I). The number and nature of admissions to hospital between cycles of treatment was noted along with the response to therapy. An observer assessment of maximal mucositis was made at the end of each cycle according to the WHO classification. Differences between glutamine and placebo supplemented treatment cycles were assessed using Student's paired *t*-test.

The serum concentration of glutamine was investigated on day 5 of the first cycle in a subgroup of ten patients. The patients were asked to fast overnight and a pretreatment serum sample obtained the following morning (09.00 h) 5 min before administration of the 4 g of glutamine or placebo supplement. A standard breakfast was given after 60 min, the chemotherapy after 150 min and lunch plus a second 4 g supplement of the same compound after 200 min. Thirteen

**Table I** Scoring system for patient-reported symptoms

| *Mouth comfort* | *Ease of eating* | *Stool consistency* |
|---|---|---|
| 1 No change from normal | 1 Eating as normal | 1 Normal stools |
| 2 Slightly sore mouth | 2 Eating is uncomfortable | 2 Soft but formed stools |
| 3 Sore mouth | 3 Pain on chewing | 3 Unformed stools |
| 4 Painful mouth with ulcers | 4 Soft food or liquids only | 4 Watery stools |
| 5 Severe pain with ulcers and inflammation | 5 Unable to eat or drink | 5 Watery and blood-stained stools |

sequential serum samples were collected over the 300 min study period. Samples were analysed for glutamine by reversed-phase high-performance liquid chromatography (HPLC) using the fluorescent method of Lindroth and Mopper (1979) and employing the modification suggested by Alfredsson *et al.* (1988). All reagents used in this assay were obtained from Sigma, Poole, Dorset, UK.

### Results

Of the 28 patients entered into the study 17 patients completed two cycles of treatment and are included in the subsequent evaluation. Of the remainder, six patients died before the second cycle was due (four glutamine, two placebo), of which four deaths were attributed to treatment toxicity (two glutamine, two placebo). At the end of the first cycle of treatment five patients had evidence of progressive disease and treatment was either changed or withdrawn (one glutamine, four placebo). At the end of two cycles of treatment one patient had achieved a partial response, 13 patients had static disease and three had evidence of progressive disease. These results from treatment reflect the advanced stage of disease in this patient group.

The supplements were virtually tasteless and well tolerated by all patients, with no apparent adverse effects. The mean (s.d.) consumption of the dose was 93% (11%) of that prescribed.

The observer assessment of oral toxicity, according to the WHO classification, is shown in Figure 1. The differences between treatment cycles were small. Overall, nine patients experienced some mucositis (WHO mucositis score $>2$) and four patients experienced severe mucositis (WHO mucositis score $\geqslant 3$) during the first cycle of treatment and eight and five patients respectively in the second cycle. The figures for treatment with and without glutamine were similar (seven and five patients with glutamine and ten and four patients with placebo).

The mean symptom scores from the diary cards for the 17 patients who completed two courses of treatment are shown in Table II. They revealed no significant difference in the severity of oral mucositis, number of bowel motions or stool consistency between glutamine- and placebo-supplemented treatments.

There was no significant difference in haematological toxicity in nine patients who had full blood counts on day 15 of each cycle (Table III). Patients were admitted to hospital between treatment cycles on only two occasions (one glutamine, one placebo).

The mean (s.d.) serum glutamine levels at time zero were 0.53 (0.06) μM for the patients receiving oral glutamine and 0.68 (0.20) μM for the placebo group. These means were not significantly different ($P = 0.3$). The time course of serum glutamine for the ten patients is shown in Figure 2. Patient 1 showed a 2-fold rise in glutamine concentration after the initial glutamine dose, which reached a peak value of 1.33 μM within 15 min and declined with a half-life of 17 min. Basal levels were achieved after 60 min. No such concomitant change in glutamine concentration was observed after lunch supplemented with glutamine. In the other four patients no definitive increase in serum concentration was detected after glutamine dosing. Minimal changes in serum glutamine levels were associated with the placebo dosing.

**Figure 1** Observer assessment of oral mucositis. **a**. Cycle 1 (▨), cycle 2 (■). **b**. Glutamine (▨), placebo (■).

**Table II** Mean ± s.d. patient-reported symptom scores

| *Symptom* | *Glutamine* | *Placebo* | |
|---|---|---|---|
| Mouth comfort | 1.56 ± 0.66 | 1.52 ± 0.62 | NS |
| Ease of eating | 1.40 ± 0.57 | 1.36 ± 0.48 | NS |
| Stool consistency | 1.87 ± 0.76 | 1.90 ± 0.81 | NS |
| No. of stools day | 1.62 ± 0.93 | 1.80 ± 0.98 | NS |

See Table I.

**Table III** Mean ± s.d. haematological indices ($n = 9$)

| | *Day* | *Glutamine* | *Placebo* | |
|---|---|---|---|---|
| Haemaglobin | 0 | 11.98 ± 1.37 | 11.88 ± 0.85 | NS |
| ($g\ l^{-1}$) | 15 | 11.06 ± 1.20 | 11.71 ± 1.19 | NS |
| | 29 | 11.64 ± 1.08 | 11.99 ± 1.31 | NS |
| WBC | 0 | 6.70 ± 3.13 | 6.98 ± 3.96 | NS |
| ($\times 10^9\ l^{-1}$) | 15 | 4.85 ± 2.53 | 4.89 ± 2.10 | NS |
| | 29 | 8.00 ± 4.67 | 7.09 ± 4.67 | NS |
| Platelets | 0 | 268 ± 123 | 263 ± 107 | NS |
| ($\times 10^9\ l^{-1}$) | 15 | 203 ± 107 | 182 ± 110 | NS |
| | 29 | 249 ± 106 | 214 ± 119 | NS |

### Discussion

The administration of this chemotherapy regimen to patients with gastrointestinal cancers was associated with similar gastrointestinal toxicity to that previously reported by Poon et al. (1989). However, in this small study the anticipated benefits of glutamine in reducing the incidence or severity of mucositis have not been fulfilled despite the strong rationale, based on previous animal studies. While this study goes against a growing body of evidence of a variety of clinical benefits attributable to glutamine or its metabolites α-ketoglutarate and ornithine ketoglutarate, it was deemed

Figure 2 Time course of serum glutamine in patients receiving oral glutamine (1–5) or placebo (6–10). Gln, Glutamine; B/F, breakfast, 5FU, 5-fluorouracil, FA, folinic acid.

inappropriate to continue with the study in its present format. Several hypotheses can be raised to account for this lack of effect which must be addressed in future studies.

In the majority of previous human studies glutamine has been administered intravenously (e.g. Ziegler *et al.*, 1990), although evidence from animal studies suggests that similar decreases in the incidence and severity of mucositis occur with enteral glutamine supplementation (Fox *et al.*, 1988). To date most studies have considered the impact of glutamine on the large bowel, and this is the first study to have specifically addressed the issue of oral mucositis. In the normal state the mucosal cells of the gut receive glutamine both from the gut lumen and from the systemic circulation. In the oral cavity the latter is the predominant source. We hypothesised that by using the glutamine solution as a mouthwash free glutamine would be made available for uptake by the cells of the oral mucosa and reduce the severity or duration of mucositis. However, this did not occur. It is possible that either these stratified squamous cells do not exhibit the preference for glutamine as a fuel demonstrated by enterocytes and colonocytes or that the duration of exposure to glutamine was too short. Pharmacokinetic studies of glutamine administration to healthy subjects have shown a significant increase in plasma gutamine concentrations with oral doses of 0.1 g $kg^{-1}$ body weight or greater (Ziegler *et al.*, 1990). No such increases were measured in a subgroup of these patients when the supplement was given following an overnight fast or with a meal. Hence, no additional benefit to the oral mucosa would be anticipated from the supply of glutamine via the systemic circulation.

The dose of glutamine given in this study (4.13 g of nitrogen) must also be considered. This dose was similar to the dose used in animals (as a proportion of total nitrogen intake) and shown to have a beneficial effect on the intestinal mucosa. However, recent human studies which have shown positive benefits have given 0.57 g of glutamine per kg, more than twice the dose in this study (Ziegler *et al.*, 1992; Schloerb & Amare, 1993), and via the intravenous route. Increasing the dose of glutamine via the oral route is not easy. The solubility of glutamine is only 3.6% at 23°C (Elia, 1992). Hence, a dose of 16 g requires over 400 ml of fluid. This is a significant burden to patients, who are often anorexic and suffer from oral mucositis.

This study differed from many others, both animal and human, in the attention given to clinical end points. Studies in laboratory animals receiving cytotoxic agents who have received glutamine-enriched diets have generally focused on the effects on the gut in terms of the maintenance of mucosal structure, and have inferred that preservation of the gut structure will lead to improvements in clinical end points. In clinical studies nitrogen balance is commonly used as a marker of clinical benefit, yet a direct relationship between the two has yet to be shown. In the study of Ziegler *et al.* (1992) in which intravenous glutamine supplementation was given to patients receiving bone marrow transplants, there was an improvement in nitrogen balance at a dose of 0.57 g $kg^{-1}$, which was not observed at the lower dose rate of 0.285 g $kg^{-1}$, but the direct clinical relevance of this is unclear. There was also a significant reduction in sepsis, shown by a decrease in the number of positive cultures, but there was no reduction in oral mucositis. In our study improvements in gut structure and nitrogen balance cannot be ruled out, but with respect to the principal clinical end point in this study, that of oral mucositis, there was no apparent effect of glutamine supplementation. Clearly, further evaluation is required of the potential role of glutamine supplementation in the management of cytotoxic-induced mucositis in terms of the most appropriate dose of glutamine, route of supplementation and clinically relevant markers of benefit. A better understanding of the mechanism of glutamine action, which is currently unclear, can only assist the rational application of this nutritional pharmacology.

We would like to thank Gail Goldberg, who supervised the randomisation of the patients in this study.

**References**

ALFREDSSON, G., WIESEL, F.A. & LINDBERG, M.J. (1988). Glutamate and glutamine in cerebrospinal fluid and serum from healthy volunteers - analytical aspects. *J. Chromatog.*, **424**, 378-384.

BERGSTROM, J., FURST, P., NOREE, L.-O. & VINNARS, E. (1974). Intracellular free amino acid concentration in human muscle tissue. *J. Appl. Physiol.*, **36**, 693-697.

CLARK, P.I. & SLEVIN, M.L. (1985). Allopurinol mouthwashes and 5-fluorouracil induced oral toxicity. *Eur. J. Surg. Oncol.*, **11**, 267-268.

ELIA, M. (1991). The inter-organ flux of substrates in fed and fasted man, as indicated by arterio-venous balance studies. *Nutr. Res. Rev.*, **4**, 3-31.

ELIA, M. (1992). Glutamine in parenteral nutrition. *Int. J. Food Sci. Nutr.*, **43**, 47-59.

FOX, A.D., KRIPKE, S.A., DE PAULA, J.A., BERMAN, J.M., SETTLE, R.G. & ROMBEAU, J.L. (1988). Effect of a glutamine-supplemented enteral diet on methotrexate-induced enterocolitis. *J. Parent. Ent. Nutr.*, **12**, 325-331.

KLIMBERG, V.S., SOUBA, W.W., SALLOUM, R.M., PLUMLEY, D.A., COHEN, F.S., DOLSON, D.J., BLAND, K.I. & COPELAND, E.M. (1990). Glutamine-enriched diets support muscle glutamine metabolism without stimulating tumour growth. *J. Surg. Res.*, **48**, 319-323.

KREBS, H. (1980). Glutamine metabolism in the animal body. In *Glutamine: Metabolism, Enzymology and Regulation*. Mora, J. & Palacios, R. (eds) pp. 319-329. Academic Press: New York.

LINDROTH, P. & MOPPER, K. (1979). High performance liquid chromatographic determination of submicromole amounts of amino acids by precolumn fluorescence derivatization with o-phthaldialdehyde. *Ann. Chem.*, **51**, 1667-1674.

MAHOOD, D.J., DOSE, A.M., LOPRINZI, C.L., VEEDER, M.H., ATHMANN, L.M., THERNEAU, T.M., SORENSEN, J.M., GAINEY, D.K., MAILLIARD, J.A., GUSA, N.L., FINCK, G.K., JOHNSON, C. & GOLDBERG, R.M. (1991). Inhibition of Fluorouracil-induced stomatitis by oral cryotherapy. *J. Clin. Oncol.*, **93**, 449-452.

NEWSHOLME, E.A., NEWSHOLME, P., CURI, R., CHALLONER, E. & ARDAWI, M.S.M. (1988). A role for muscle in the immune system and its importance in surgery, trauma, sepsis and burns. *Nutrition*, **4**, 261-268.

O'DWYER, S.T., SCOTT, T., SMITH, R.J. & WILMORE, D.W. (1987). 5-Fluorouracil toxicity on small intestinal mucosa but not white blood cells is decreased by glutamine. *Clin. Res.*, **35**, 367A.

POON, M.A., O'CONNELL, M.J., MOERTEL, C.G., WIEAND, H.S., EVERSON, L.K., KROOK, J.E., MAILLIARD, J.A., LAURIE, J.A., TSCETTER, P. & WIESENFELD, W. (1989). Biochemical modulation of fluorouracil: evidence of significant improvement of survival and quality of life in patients with advanced colorectal carcinoma. *J. Clin. Oncol.*, **7**, 1407-1418.

SCHLOERB, P.R. & AMARE, M. (1993). Total parenteral nutrition with glutamine in bone marrow transplantation and other clinical applications (a randomised, double-blind study). *J. Parent. Ent. Nutr.*, **17**, 407-413.

SMITH, R.J. (1990). Glutamine metabolism and its physiologic importance. *J. Parent. Ent. Nutr.*, **14**, 40S-44S.

WINDMUELLER, H.G. & SPAETH, A.E. (1985). Uptake and metabolism of plasma glutamine by the small intestine. *J. Biol. Chem.*, **249**, 5070-5079.

ZIEGLER, T.R., BENFELL, K., SMITH, R.J., YOUNG, L.S., BROWN, E., FERRARI-BALIVIERA, E., LOWE, D.K. & WILMORE, D.W. (1990). Safety and metabolic effects of L-glutamine administration in humans. *J. Parent. Ent. Nutr.*, **14**, 137S-146S.

ZIEGLER, T.R., YOUNG, L.S., BENFELL, K., SCHETLINGA, M., HORTOS, K., BYE, R., MORROW, F.D., JACOBS, D.O., SMITH, R.J., ANTIN, J.H. & WILMORE, D.W. (1992). Clinical and metabolic efficacy of glutamine-supplemented parenteral nutrition after bone marrow transplantation. A randomised, double-blind trial. *Ann. Int. Med.*, **116**, 821-828.